# The effectiveness of empathy training in health care: a meta-analysis of training content and methods

**DOI:** 10.5116/ijme.61d4.4216

**Published:** 2022-01-26

**Authors:** Christoph M. Paulus, Saskia Meinken

**Affiliations:** 1 Faculty of Empirical Human Sciences and Economics, Saarland University, Germany

**Keywords:** Empathy training, meta-analysis, effectiveness, training content, training methods

## Abstract

**Objectives:**

The meta-analysis examined the question of whether empathy training is effective in health care and whether specific training content and methods can be found to account for its effectiveness.

**Methods:**

We included 13 out of 50 studies (total N = 1315) that fulfilled the search criteria. R version 4.0.5 with the esc, meta, metafor, and dmetar packages and SPSS28 were used to conduct the meta-analysis based on the random-effects model. The effect sizes were calculated using Hedge`s g, and heterogeneity was tested using Cochran's Q. In addition, the multicollinearity of the moderators was checked.

**Results:**

The overall effect size (Hedge´s g = 0.58, s = 0.10, p = 0.00) indicated a moderate effect of empathy training. There was a significant heterogeneity (I^2 ^= 76.9%, Q = 84.82, p=0.00), thus we examined whether individual training methods have influenced effect sizes, which could not be confirmed (F _(8,4)_ = 0.98, p = 0.55). The same applied to the training contents (F _(6,6)_ = 0.27, p = 0.93).

**Conclusions:**

The present study showed that empathy training could be effective. This confirmed previous findings and supported the use of such training. However, according to our results, no significant moderators could be found, i.e., the training contents or methods did not contribute to the effect sizes. For meaningful findings, a comparison of different training components should definitely be made, and it should be investigated whether empathy training spread over a period of time is more effective and sustainable than one-time training.

## Introduction

Empathy is the ability to understand and share the internal state of others with the consequence of being able to respond appropriately to it.^1^ Empathy is, therefore, a process directed at the emotional responses of others, which includes an emotional response of one's own.^2^ An important prerequisite for this is the ability to adopt perspectives, which is a basic component in all empathy theories.^3^^,^^4^ Cognitive perspective-taking refers to the ability to understand the thoughts and feelings of a counterpart and predict their behavior and reaction.

The ability to decenter is seen as part of social and cognitive development and is therefore age-dependent. Between the ages of three and eight, children have only undifferentiated assumptions about the thoughts and motives of other people. They do not distinguish between external behavior and internal drives. At this early age, they can already recognize the basic emotions of fear, sadness and joy from facial expressions,^5^ but they do not yet distinguish between their own and others' reactions in certain situations. The ability to see oneself from another perspective develops only at the age of seven to twelve, accompanied by the insight that emotions can also be feigned or that competing experiences (e.g., curiosity and insecurity) can also occur in parallel. The highest level, according to Selman,^6^ is the social-symbolic perspective-taking and describes the recognition that not all motives and emotions can be accessed self-reflexively and relationships between people can exist on multiple levels (superficial to deeper ones).^7^ Consideration of age is essential because, when examining empathy training for effectiveness, as described below, there may be significant age differences between trainees and practitioners in medical professions that could influence effect sizes.

Empathy and perspective-taking correlate positively with agreeableness and openness^8^ and are considered to be an important influencing factor for coping with social demand situations,^7^ as they occur time and again in the medical context. Especially in medical professions, empathy is a necessary quality for effective patient care.^9^ In the context of health care, empathy is understood as a predominantly cognitive skill that focuses on the understanding of the patient's situation and the ability to communicate this understanding.^10^ Thus, patients rate physicians' empathy as very important in discussions with doctors.^11^ In addition, there is a positive correlation between physicians' empathy and patients' treatment outcome.^12^ Physicians who can also correctly interpret non-verbal cues from patients can better build trust and reduce anxiety.^13^ However, this circumstance also helps physicians to strengthen their effectiveness and professional satisfaction, and therefore also prevents, among other things, burnout.^14^ On the other hand, however, the literature criticizes physicians for often lacking empathy, being too detached, and for being dispassionate in their dealings with patients.^15^ In addition to this, a decrease in empathy is observed during medical school.^16^ As a result, empathy training is needed not only to promote empathy in general but also to prevent empathy deficits. Thus, empathy training for medical personnel has also been considered as an official learning goal of the Association of American Medical Colleges since 1998.

Nonetheless, it can be objected that the promotion of empathy in terms of the affective component is not exclusively recommended in the context of health care. This is because, in a professional application, it is essential that professionals maintain a certain degree of emotional distance to avoid inappropriate emotional involvement of the professional and consequent personal emotional exhaustion. In addition, objectivity leads to an appropriate professional response and patient-centered communication.^10^ If these two aspects are considered, it can be assumed that inpatient care, the empathy of professionals is always beneficial.

Consequently, if empathy training is effective in the context of healthcare, there should be an improvement in cognitive empathy or behavioral empathy.^17^

It is known that there are by now many approaches to empathy training in the context of medical education, but rarely any commonalities in these training regarding the concept of empathy, the training contents, the training methods, the age of the subjects or the duration of the training.

Typically, a meta-analysis across studies is a good way to find the effects of these variables. In recent literature since 2016, there have been two meta-analyses that have looked at the trainability of empathy^15^^,^^18^ Below, the results of these studies are briefly prediscussed.

Teding van Berkhout and Malouff^18^ demonstrated a medium effect (Hedge`s g = .51, adjusted for estimated publication bias after trim-and-fill analysis) for the overall effectiveness of empathy training programs. After excluding one outlier study that showed a very large effect in a small number of participants, the meta-analysis included^18^ randomized controlled trials of empathy training with a total of 1018 participants. Participants included university students and health professionals, as well as patients, other groups of adults, adolescents, and children. The results of the moderator analysis indicated that the training worked best with health professionals and university students who were compensated for their time and received training in cognitive and behavioral empathy. In this regard, studies of empathy training that included all four components of behavioral training (instruction, modeling, practice, and feedback) did not have significantly larger effect sizes than other studies. Furthermore, the number of training hours and the time between pre-and post-intervention assessment were not statistically significantly associated with the effect size. The results indicated that experimental research on the effects of different types of participants, training conditions, and assessment types are needed.

The meta-analysis by Fragkos and Crampton^15^ summarized the various results of randomized controlled trials of clinical empathy interventions for medical students. The results of the meta-analysis showed that empathy interventions for medical students significantly increased students' empathy compared with control groups. The pooled standardized mean difference SMD = .68 indicated a medium positive effect. Sixteen studies were included in the analysis, and the total number of participants was 1736. A number of moderating variables, such as age, country, the extent of empathy measurement, type of empathy intervention and presence of a sample, influenced the effectiveness of these interventions.

In contrast, the same analysis´ nonsignificant moderators were gender, study quality, journal influence, and training characteristics, such as duration, control type, effect duration, and compensation. The authors found out that the behavioral modeling component of empathy interventions was more effective in developing empathy when practice was present and mixed-model interventions (for example, experiential education, didactics and skills training) were used. Based on these results, it was thus suggested that training should primarily involve exercises that incorporate a mixture of pedagogical techniques (experiential, didactic, and skills training) and conduct training on students toward the end of their medical studies. In addition to this, empathy should be objectively assessed by experts or standardized patients. Furthermore, the definition of empathy should be broad enough to include multidimensional elements (cognitive, affective, and behavioral).^15^

There are by now many effective approaches to empathy training in medical education from many different countries, e.g., Italy,^19^ USA,^20^ Brazil,^21^ Iran,^22^ and Spain.^23^ But there is no comprehensive analysis of whether individual training methods, such as role-play or feedback, might moderate such effects. Additionally, there is also a lack of knowledge about training content, such as perspective-taking or self-reflection. Such an analysis could help to design particularly efficient empathy training since it allows to weigh the effort and effectiveness of individual training components. The findings could thus serve as a basis for developing further training programs.

These research questions will be examined in this meta-analysis: Does empathy training increase empathy in health care professionals? Are there differences in the outcomes (effect sizes) of empathy training? What moderator variables contribute to these effect sizes?

## Method

The search for appropriate studies took place using key terms in electronic databases. Databases were searched via online portals (Google Scholar, PubMed, and Web of Science) using keywords deemed relevant to this work, "Empathie, Empathie Training, empathy, empathy training nurses, empathy training physicians, empathy training residents, empathy training medical personnel". Keywords were initially determined by using the search terms from existing meta-analyses on empathy training, with adjustment for the population. Additional keywords were selected based on the indicated keywords of the already included studies. In addition to this, the so-called "grey literature" was considered. Studies suitable for meta-analysis were preselected based on title and abstract, narrowed down more specifically, and examined more closely for the following inclusion criteria. If data was missing, authors were contacted.

R version 4.0.5 with the esc, meta, metafor, and dmetar packages and SPSS 28 were used to conduct the meta-analysis. The meta-analysis in this paper is based on the random-effects model, as this is the recommended model to use in clinical psychology and health sciences.^25^ This is because, in contrast to the fixed-effects model, the random-effects model assumes that effect sizes vary not only based on sampling error but also based on differences between studies, for example, due to differences in participants or study designs. The significance level for all calculations was 0.05 unless otherwise stated.

### Inclusion criteria

The studies collected in the literature search were reviewed for the following criteria:

     1)The aim of the study was to investigate empathy training for the general promotion or prevention of empathy deficits. Training programs in an occupational context are designed for the general promotion or prevention of empathy deficits. These training programs are qualitatively different from studies with training targeting, for example, clients with identified empathy deficits.^17^ To ensure comparability across studies, the studies' training must have the same or similar goals.

     2) The study population consisted of these health care professionals: physicians, medical students, or nurses.

     3)The study was an experimental or quasi-experimental study. Only primary studies that contained quantitative empirical information are included in the analysis. This empirical information could be either an effect size or data that could be used to calculate an effect size. To ensure the quality of the studies, appropriate study designs were considered to be experimental designs or quasi-experimental designs. These include, for example, pre-post and intervention/control group designs.

     4) Empathy was measured in the study. For which either self-report scales, observational scales, knowledge tests, or combinations of these were used. No further restrictions were made.

     5) The training (including training methods and content) was recorded. Individual training components are of central importance, as they can provide information about which specific training methods and contents are most effective. For this reason, these training components had to be recorded in the primary studies in order to be able to examine them more closely in a moderator analysis.

     6) The study was available in English or German and was published after 2008.

### Exclusion of studies

During the search of the databases, studies were already excluded on the basis of title and abstract that did not meet one or more of the previously mentioned inclusion criteria. In addition, studies with nonsignificant results were excluded. Studies that were not available in full text got also excluded. Of the approximately 50 remaining full-text articles, a total of 13 relevant studies could be used for this meta-analysis after reviewing the inclusion criteria.

### Coding scheme

The studies were analyzed using a coding scheme on the one hand at the effect size level and on the other hand at the study level. At the effect size level, the statistical information given in the studies, such as pre- and post-mean values and standard deviations of the groups, or the given effect sizes, which are necessary for calculating and correcting the effect size, was coded.

At the study level, the sample, with sample size, mean age in years, and percentage of female participants were coded. In addition, the year of publication and country of the study, the target group of the training, the components of empathy taught in training, the measures of empathy measured in the studies, and the total hours of training were coded.

In this meta-analysis, the focus is on coding the training methods and content. The reported training methods are shown in Table 1, and the training contents are described in Table 2.

**Table 1:** Coding of the training methods

Method	Definition	Example
(1) Role play	A training method that involves actively trying out behaviors in simulated situations.	Test behavior in a difficult situation with simulated patients.
(2) Discussion	Exchange of ideas on specific topics in the training group.	Sharing the pros and cons of putting yourself in the patient's shoes.
(3) Feedback	The feedback that the training participant receives from the training instructor or from the group to compare self-image and external image.	Feedback on how the behavior shown is received and what potential for improvement is identified.
(4) Lecture	Information transfer offered by the trainer in the context of frontal teaching.	Presentation of the trainer about the theory of empathy.
(5) Elaboration of a topic in individual work	Tasks or exercises that the training participant works on alone and in which he or she acquires knowledge independently.	Silent work in which the participant reflects on a situation by himself.
(6) Elaboration of a topic in a team	Tasks or exercises that the training participant works on in a group or in partner work to acquire knowledge as a team.	In partner work, rewrite a failed dialogue.
(7) Conversation with experts	Knowledge acquisition in the context of a conversation with experts, i.e., people who are particularly knowledgeable in a field.	Question and answer session with the empathy expert.
(8) Observation of exemplary interactions	All methods in the sense of model learning. This also includes the use of digital media.	Observation of a patient-doctor interaction using videos.

**Table 2:** Coding of the training contents

Content	Definition	Example
(1) Communication and its challenge: theory and conversation guide	All training content that deals with the topic of communication. This includes all concepts and theories of communication, barriers and problems in communication processes and conversation guidelines and communication guides.	Elaboration of the 6 steps of the Buckman Protocol: how to deliver bad news.
(2) Self-reflection and learning from one's own experience	The mindfulness, the recognition and perception of one's own feelings and emotions, and all the reflections that the participant makes or has made about his own situation and himself.	Reflection on a situation in which the participant himself did not feel understood by others.
(3) Importance of empathy	Any content that emphasizes the importance of empathy.	Trainer's statement: "There is a positive correlation between clinician empathy and patient outcomes."
(4) Theory of empathy	All theories and concepts taught on the topic of empathy in general.	"Empathy has a cognitive, affective, ... component."
(5) Adopting perspective and learning from the experience of others	All content in which participants put themselves in the shoes of others and all reflections the participant makes or has made about the other person's situation.	Reflection on a situation in which someone else might not have felt understood.
(6) Empathic behavior	Training content in which understanding is expressed and empathy is shown.	Say to the patient, "This situation must be very difficult for you."

To ensure the objectivity of coding, all training components (76 cases for method and 89 cases for content) were coded by two independent raters. Rater agreement for training method was very high (κ = 0.91, p = 0.001). Agreement for training content was also good (κ = 0.89, p = 0.001). The remaining disagreements were resolved by a brief discussion.

### Overview of primary studies and sample description

Based on the literature search and the aforementioned selection criteria, 13 eligible studies were identified for inclusion in the meta-analysis. All participants in the studies were healthcare professionals or prospective healthcare professionals. The total sample yielded N = 1315 participants with a mean age of 28.33 years and a 58.54% proportion of female participants. A detailed description of the studies can be found in the Appendix.

### Effect sizes

The effect sizes of the primary studies were calculated using the relatively conservative Hedge`s g. Although the statistics of Cohen`s d and Hedge`s g are similar, Hedge`s g is preferred to Cohen`s d because Hedge`s g reduces the error of the estimate by a slight modification in the calculation of the pooled variance, especially when the sample size is small (n < 20).^24^ The effect sizes of the studies with corresponding confidence intervals were calculated on the basis of the statistical information reported in the studies, such as pre-and-post means and standard deviations of the groups or even the reported effect sizes. If a study had multiple relevant outcomes, the mean of the relevant effect sizes was taken as the study effect size to avoid overweighting the study. All effect sizes were summarized by the random-effects model described previously to obtain an overall effect size estimate. The goal is to obtain the mean distribution of true effects,^25^ and the individual effect sizes were weighted by their respective standard errors.

### Heterogeneity

Heterogeneity of effect sizes was tested using Cochran's Q, because it "is considered a most reliable indicator of heterogeneity in meta-analyses".^26^ With a significant Q statistic, the study effect sizes are different enough to assume that the overall effect size is not a good estimate of the overall effect, or that the effect is moderated. In addition to this, I^2^ was calculated to quantify the heterogeneity present. Twelve is easier to interpret than Cochran's Q and can be interpreted as the ratio of between-study variance to the total variance. The measure is fixed between 0% and 100%. Above an I^2^ of 75%, significant heterogeneity must be assumed.^26^

Heterogeneity can also be caused by outliers with particularly large or particularly small effect sizes. This can be due to the fact that the quality of the study is low, or the sample is very small. Since outliers distort the overall effect size, it is sometimes useful to remove them from the analysis and then perform a new calculation to check whether a change could be observed or not.

### Moderator analysis

Heterogeneity provides a reason to conduct a moderator analysis to identify the variables that influence the variance in effect sizes. The possible moderator variables were selected with respect to the development of empathy training. Accordingly, the primary focus was on training methods and content. For this purpose, regression analytic procedures (multiple meta-regression) were used. R^2^ was used to test the fit of the meta-regression model. R^2^ quantifies the heterogeneity variance of the model in relation to the total heterogeneity in percentage.^25^ A high percentage is associated with a high fit of the model. In addition, the multicollinearity of the moderators was checked to ensure that only variables that are independent of one another are used. In this work, the investigation of multicollinearity, meta-regression, and model fit testing was performed using the metafor package.

### Publication bias 

Potential publication bias was evaluated using the funnel plot technique. This visualizes whether small studies with small effect sizes are missing. If there is no publication bias, all studies, sorted by their effect sizes and standard errors, would be symmetrical in the form of a funnel in the diagram. The "trim-and-fill" method is also based on the funnel plot and, in addition to detection, aims to correct publication bias.^25^ To restore symmetry in the case of publication bias, studies that cause asymmetry are removed, and missing studies are artificially added.

## Results

### Integration of effect sizes

**Figure 1:**
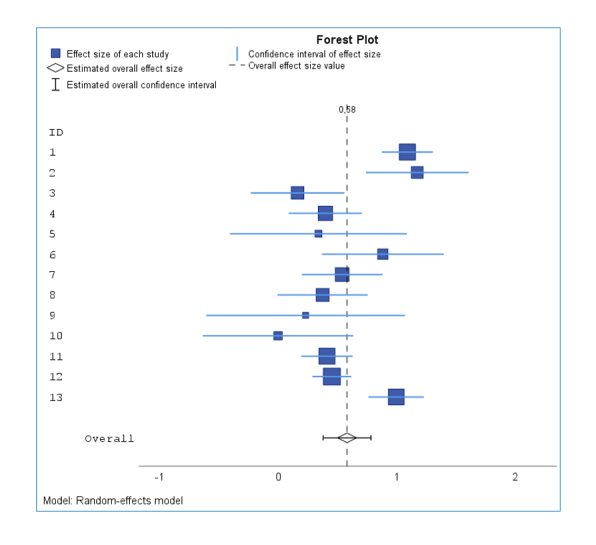
Forest Plot including the overall effect size value

**Figure 2:**
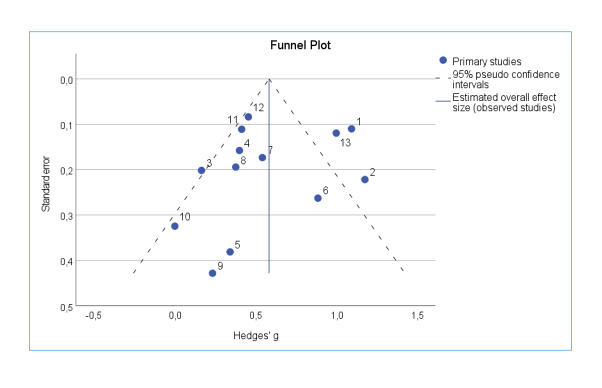
Funnel Plot with pseudo confidence intervals and estimated overall effect size

The results of the effect size calculations are shown in the forest plot in Figure 1, and the funnel plot in Figure 2, effect sizes per study are shown in the Appendix. Numerically larger effect sizes mean greater effectiveness of empathy training. The training seemed to have had at least a small effect in all studies.

The overall effect size (Hedge´s g = 0.58, s = 0.10, Z = 5.98, p = 0.00) for the random-effects model, with all 13 studies included, became statistically significant. This means that empathy training is highly likely to promote empathy. The analysis showed significant heterogeneity in the individual effect sizes (Q = 84.82, p = 0.00). However, the relative size I^2^ of 76.9% indicates that the magnitude of heterogeneity is high because there are large differences in study outcomes, and the overall effect size may not be a good estimate of the overall effect or may even be moderated. Two outliers were found

with substantial effect sizes. The confidence intervals of studies 1 (g = 1.09 [0.88; 1.31])^27^ and 2 (g = 1.51 [1.24; 1.78]) ^23^ did not overlap the confidence interval of the overall effect. Without the two outliers, the overall effect size was Hedge's g = 0.51 with a confidence interval of [0.39; 0.62]. Even after excluding the outliers, the heterogeneity test became significant (Q = 19.46, p = 0.03), with the extent of heterogeneity having de-creased to "moderate" (I^2^ = 48.6% [.0%; 74.3%]). Additionally, the quality of the two studies has already been reviewed in the inclusion criteria and was considered to be ad-equate. On the one hand, high power can be assumed in study 1^27^ because the study had a high sample number (n = 190); on the other hand, the high effect size of the study (Hedge´s g = 1.09, s = 0.11) might have resulted from the fact that training content and measures of empathy matched exactly in content. Study 2^23^ was also considered reliable because the study had multiple instruments measuring empathy.

These explanations were based on content dimensions and were subjective, but the examination of publication bias also indicated that the overall effect size was not overestimated. Thus, it was deemed unnecessary to exclude the two studies identified as outliers.

### Moderator analysis

The multicollinearity test did not find high correlations between the moderator variables, so no variable was excluded. First, we examined whether individual training methods influenced effect sizes, which was not confirmed (F _(8,4)_ = 0.98, p = 0.55). Therefore, further investigation of the fit of the model was refrained from (See Table 3).

**Table 3:** Regression coefficients of training methods (all nonsignificant)

Training methods	Regression coefficients	SD^*^	LC^**^ interval	HC^***^ interval
Intercept	0.52	0.46	-0.77	1.82
Role play	0.24	0.29	-0.56	1.05
Feedback	0.17	0.31	-0.71	1.04
Individual work	0.17	0.27	-0.57	0.92
Observation	0.11	0.38	-0.94	1.17
Teamwork	0.04	0.26	-0.68	0.76
Discussion	-0.13	0.33	-1.05	0.78
Lecture	-0.28	0.34	-1.25	0.68
Conversation with experts	-0.28	0.25	-0.97	0.41

*Standard deviation: **Low confidence; ***High confidence

The examination of whether the individual training contents moderate the effectiveness of the training was also not significant (F _(6,6)_ = 0.27, p = 0.93). Thus, no moderating training content can be identified either (See Table 4).

## Discussion

The aim of the meta-analysis was to examine the effectiveness of empathy training tailored to healthcare professionals and to clarify whether the training components (method and content) moderate the effect.

**Table 4:** Regression coefficients of training content (all nonsignificant)

Training content	Regression coefficients	^*^SD	^**^LC interval	^***^HC interval
Intercept	0.25	0.52	-1.03	1.54
Communication	0.26	0.44	-0.81	1.34
Importance of empathy	0.21	0.41	-0.76	1.21
Behavior	0.15	0.48	-1.02	1.33
Self-reflection	0.08	0.31	-0.67	0.85
Adopting perspective	0.05	0.32	-0.74	0.85
Theory	-0.27	0.32	-1.06	0.52

*Standard deviation: **Low confidence; ***High confidence

The first research question examined whether empathy training can increase empathy in healthcare professionals. The result of this meta-analysis indicated a moderate effect of empathy training. Therefore, it can be concluded that empathy training increases empathy in health care professionals, and its use should be considered reasonable. The result is consistent with findings from previous meta-analyses on empathy training.^15^^, ^^17^^, ^^18^

Furthermore, the second research question analyzed to what extent there are differences in the results, respectively, between the effect sizes of empathy training. The heterogeneity in the meta-analysis was high (I^2^ = 76.9%), which indicates that there are differences in the results of empathy training and could be due to the differences in the implementation of the individual training, as there are many possibilities to design training programs to increase empathy.^17^

In the context of the third research question, training components such as training methods and content were examined as possible modifiers for the first time in the present analysis. No significant moderators could be found, i.e., the training components did not contribute to the effect sizes. Due to the small number of studies, no moderator interactions could be calculated, as the number of parameters to be estimated was larger than the number of observations.^25^ In addition, when the number of studies is small, the power of the moderator analysis decreases so that large differences in results could be identified as statistically significant. It has been pointed out that nonsignificant trends, however, provide evidence for the existence of possible moderators.^18^

The regression coefficients for the training contents "communication" and "role play" were estimated as the highest ones in the meta-regression model. The effectiveness of the method "role play" in training could already be proven for training in general.^28^ Fragkos and Crampton^15^ have also shown that practice of what is learned seems to be significant for the effectiveness of empathy training. This is because active learning increases the likelihood that what is learned will be retained and applied later.^28^

The training component "communication" is also important for empathy training tailored to healthcare professionals. This is because the professional must be able to successfully communicate to the patient, for example, that they understand the patient's situation. For this reason, the component of empathy that is trained is of great interest, too. In 12 of the 13 included studies, the behavioral component was trained in addition to the cognitive component, and only in 7 studies was the affective component trained. This supports the knowledge that empathy training often train cognitive and behavioral component and less the affective component.

In contrast to previous meta-analyses, current studies were included, whose training processes are described in detail. Thus, for the first time, a comprehensive analysis of individual training methods and training content could be made by investigating whether individual training components moderate training effects. The difference between the study of Teding van Berkhout & Malouff^18^ and the current study is that the meta-analysis of Teding van Berkhout & Malouff captures findings on the broad population. In contrast, this study focuses on the effectiveness of empathy training to promote empathy in general or to prevent empathy deficits tailored to healthcare professionals and prospective healthcare professionals. The Fragkos & Crampton^15^ meta-analysis has in common with this work that the focus was on studies with training for the general promotion and prevention of empathy deficits. However, Fragkos & Crampton's work focused exclusively on medical students and the results of randomized controlled trials.

## Conclusions

Empathy training can certainly be effective. However, no adequate answer can be given to the question posed in the introduction about what constitutes effective empathy training. The moderator analysis in this study aimed to determine which training components influence the effectiveness of empathy training. Fragkos and Crampton^15^ have already provided evidence that a mixture of methods (experiential, didactic and skills training) could be crucial. Regarding the relationship between training methods and content, it can be stated that some training methods are per se determined by training content. For example, the content of the communication is often taught with role play^22^ or self-reflection in combination with the elaboration of a topic in individual work.^29^ Moreover, it cannot be ruled out that other training components exist that would be conducive to effective empathy training. For example, the component homework was hardly found in any of the training of the analyzed studies. However, homework could be important in the development of training because it involves practicing what has been learned, which is crucial according to Fragkos and Crampton.^15^ Thus, it should be investigated whether homework increases the effectiveness of training or not.

For meaningful findings, a comparison of different training components and their variation should definitely be made. With regard to the development of scientifically based empathy training, it is also of interest to determine more precisely the balance between economically efficient training and effective training. For example, short, one-time training with large groups is of interest from an economic perspective. This meta-analysis found considerable variability in the number of hours and group sizes of the studies. The training times of the studies in this meta-analysis varied from 2 hours as compact training to 20 hours of total training time spread over weeks. Short, one-time training is far more economical, but this could be associated with a loss of efficacy. Therefore, it should be investigated whether empathy training spread over a period of time is more effective and sustainable than one-time training.

### Limitations

The included studies focused on empathy as the object of measurement. The fact that the outcome variable empathy was measured using different instruments limits the comparability of the studies. This is because different measurement instruments of empathy also tend to yield different expressions of the construct. For example, a person may score high on the Neurobiology and Physiology of Empathy test, but at the same time, score low on the Interpersonal Reactivity Index (IRI). However, if the measurement instruments had been more strictly delimited, the number of studies used would have been insufficient. Moreover, in 10 of 13 studies, only one instrument was used, which could have a negative impact on the reliability of the study results.

The low number of included studies can be explained by the quality and documentation of many studies. For example, a large proportion of the studies lacked either statistical characteristics, the training program, or training components in the initial selection in order to be able to examine them in the context of a moderator analysis. Even studies that could be included limited the validity of the result due to the different presentation and inconsistent protocol. For example, many studies lacked information on the theory on which the training was based, information on the detailed procedure of the training itself (including the sequence of training components and definitions of these), information on the sample, the study design, and the results with all characteristic values. The varying detail of the data caused difficulties in coding and thus reduced the comparability of the studies. Consequently, a limitation of this meta-analysis was that studies were grouped together even though they had differences in the instruments used to measure empathy or differences within the population of health professionals, in addition to the different training themselves. Smith and Norman^20^ counter that the resulting heterogeneity may interest a moderator analysis.

Another limitation could be that too many moderator variables were included for too few studies. Thus, as mentioned above, no moderator interactions could be calculated. Furthermore, overfitting of the meta-regression model can be considered. To prevent this, the choice of moderators should be minimized based on predefined scientific or theoretical questions and defined before the start of the study. In the context of this work, however, these guidelines could not be followed because no training components have been investigated or compared in the context of empathy training to date. It was only possible to fall back on general training methods that had already been studied,^28^ and the moderator variables had to be extracted from the studies themselves. However, the low multicollinearity argues against a reduction of the moderator variables.

Finally, for the development of effective training, it would be appropriate to pay attention to the different target groups within the population of healthcare professionals as well. The target groups in the studies of the present meta-analysis varied from medical students to nursing students and residents to experienced professionals. It could be considered that already experienced professionals should be trained differently than job entrants or trainees. This investigation could also take place through a combination of different training with different groups.

### Acknowledgements

We acknowledge support by the Deutsche Forschungsgemeinschaft (DFG, German Research Foundation) and Saarland University within the funding program Open Access Publishing.

### Conflict of Interest

The authors declare that they have no conflict of interest.

## Supplementary material

Supplementary file 1 Appendix. Overview of the studies included in the meta-analysis
