# Supplementary material for: The effectiveness of empathy training in health care: a meta-analysis of training content and methods
Source: Int J Med Educ. 2022 Jan 26;13:1–9. doi: 10.5116/ijme.61d4.4216 (PMC8995011; doi:10.5116/ijme.61d4.4216)
Supplement: Supplementary file 1 — Appendix. Overview of the studies included in the meta-analysis [file ijme-13-1-S1.pdf]

Appendix

Overview of the studies included in the meta-analysis

Author (year)	Method	Training content	Effect Size (Std. Error)	Subjects	Sample size	Mean age	Ratio of Female in %
1. Ancel (2006)[27]	1, 2, 3, 4, 5, 8, 9	1, 2, 3, 5, 6	1.09 (0.11)	Nurses	190	ca 26.07	n.i.
2. Bas-Sarmiento, Fernández-Gutiérrez, Baena-Baños, Romero-Sánchez (2017)[23]	1, 3, 5, 6, 8	1, 3, 4	1.18 (0.22)	Nursing students	48	23.04	79.16
3. Cunico, Sartori, Marognolli & Meneghini (2012)[19]	1, 2, 3, 4, 5, 6, 8	1, 2, 3	0.16 (0.20)	Nursing students	103	n.i.	73
4. D'souza, P., Rasquinha, D'souza, T., Jain, Kulkarni & Pai (2019)[13]	1, 4, 5, 8	1, 3, 4, 5, 6	0.40 (0.16)	Medical students	82	19.49	49.44
5. Esfahani, Behzadipour, Nadoushan, Shariat (2014)[31]	1, 2, 4	1	0.34 (0.38)	Residents for psychiatry	14	30.35	71,43
6. Gholamzadeh, Khastavaneh, Khademian & Ghadakpou (2018)[22]	1, 2, 4, 5, 7, 8	1, 2, 3, 4, 5, 7	0.88 (0.26)	Nursing students	63	22.7	55.6
7. Kataoka, Iwase, Ogawa, Mahmood, Sato, DeSantis, Hojat & Gonnella (2018)[32]	1, 2, 3, 4, 9	1	0.54 (0.17)	Medical students	69	n.i.	39
8. Riess, Bailey, Dunn & Phillips (2012)[34]	2, 4, 5, 8	1, 2, 3, 5	0.38 (0.19)	Residents	99	30.6	52
9. Riess, Kelley, Bailey, Konowitz & Tutt Gray (2011)[33]	2, 4, 5, 6, 8	1, 2, 3, 4, 5, 6	0.23 (0.43)	Residents for otorhinolaryngology	11	31	43
10. Sands, Stanley & Charon (2008)[29]	2, 5	2, 6	0.00 (0.32)	Pediatric oncology professionals	19	41.1	n.i.
11. Schweller, Ribeiro, Celeri & De Carvalho Filho (2017)[21]	2, 3, 5, 7, 8	1, 3, 6	0.41 (0.11)	Medical students	166	n.i.	n.i.
12. Williams, Brown, McKenna, Palermo, Morgan, Nestel, Brightwell, Gilbert-Hunt, Stagnitti, Olaussen & Wright (2015)[35]	6, 7, 8	3, 4, 5, 6	0.45 (0.08)	Students of medicine and health professions	293	n.i.	77.1
13. Wündrich, Schwartz, Feige, Lemper, Nissen & Voderholzer (2017)[30]	1, 2, 3, 8, 9	1, 2, 3, 6	1.00 (0.12)	Medical students	158	n.i.	n.i.

Note: If no information was given in a study, this is indicated by "n.i.", cod¬ing of training methods are described in Table 1, coding of training contents are described in Table 2.
